# Targeting VEGF, PARP, and FRα Pathways in Ovarian Cancer: Clinical Advances with Bevacizumab, Rucaparib, and Mirvetuximab Soravtansine

**DOI:** 10.3390/jcm15051742

**Published:** 2026-02-25

**Authors:** Piotr Kawczak, Tomasz Bączek

**Affiliations:** 1 Department of Pharmaceutical Chemistry, Faculty of Pharmacy, Medical University of Gdańsk, 80-416 Gdańsk, Poland; tomasz.baczek@gumed.edu.pl; 2 Department of Nursing and Medical Rescue, Institute of Health Sciences, Pomeranian University in Słupsk, 76-200 Słupsk, Poland

**Keywords:** ovarian cancer, bevacizumab, rucaparib, mirvetuximab soravtansine, VEGF, PARP, FRα, targeted therapy, gynecologic malignancies, precision oncology

## Abstract

Ovarian cancer remains a leading cause of gynecologic cancer–related mortality worldwide, and long-term outcomes with conventional cytotoxic chemotherapy remain limited. The integration of targeted therapies has substantially reshaped treatment paradigms by exploiting key molecular pathways involved in angiogenesis, DNA damage repair, and folate receptor signaling. This review synthesizes evidence from pivotal phase II and III clinical trials, translational studies, and meta-analyses evaluating inhibition of the VEGF, PARP, and folate receptor-alpha (FRα) pathways, with a focus on bevacizumab, rucaparib, and mirvetuximab soravtansine. Across disease settings, these agents have demonstrated clinically meaningful improvements in progression-free survival, durability of response, and tolerability when deployed in biomarker-selected populations. Bevacizumab has shown consistent benefit when combined with platinum-based chemotherapy and as maintenance therapy in advanced disease. PARP inhibitors, including rucaparib, exploit homologous recombination deficiency (HRD) to induce synthetic lethality and are now central to frontline and recurrent treatment strategies for BRCA-mutated and HRD-positive tumors. Mirvetuximab soravtansine has emerged as an effective and well-tolerated option for patients with FRα-high, platinum-resistant ovarian cancer, addressing a longstanding unmet clinical need. Collectively, VEGF-, PARP-, and FRα-targeted therapies have enabled more rational treatment sequencing, informed combination strategies, and personalized clinical decision-making in ovarian cancer. Ongoing efforts to define optimal sequencing, overcome acquired resistance, and refine predictive biomarkers are expected to further enhance the durability and breadth of benefit from targeted therapies and advance precision oncology in gynecologic malignancies.

## 1. Introduction

Ovarian cancer remains one of the most challenging malignancies in gynecologic oncology, accounting for a disproportionate burden of cancer-related mortality among women worldwide despite significant advances in surgical techniques and systemic therapies [1,2,3,4]. Epithelial ovarian carcinoma (EOC), which comprises approximately 90% of ovarian malignancies, carries the highest mortality rate among gynecologic cancers, largely because most patients present with advanced-stage disease. This delayed diagnosis reflects the nonspecific nature of early symptoms and the persistent lack of an effective population-based screening strategy capable of reliably detecting early-stage disease [5,6,7]. Even among patients who undergo optimal cytoreductive surgery followed by first-line platinum-based chemotherapy, recurrence is common, and many ultimately develop platinum-resistant disease—an inflection point that markedly diminishes the efficacy and durability of conventional cytotoxic treatments [8,9,10].

Ovarian cancer is a biologically heterogeneous disease encompassing multiple histologic and molecular subtypes, each characterized by distinct patterns of behavior, prognosis, and therapeutic vulnerability. High-grade serous carcinoma (HGSC), the most common and lethal subtype, is defined by near-universal TP53 mutations, frequent defects in homologous recombination DNA repair—including BRCA1/2 mutations—and extensive genomic instability. These molecular features underlie both the relative chemosensitivity of HGSC and its susceptibility to DNA repair–targeted therapies. In contrast, low-grade serous carcinoma (LGSC) follows a more indolent clinical course, is frequently driven by MAPK pathway alterations such as KRAS and BRAF mutations, and demonstrates limited responsiveness to conventional chemotherapy but potential sensitivity to hormonal and MAPK-directed therapies. Endometrioid ovarian carcinoma, often associated with endometriosis, commonly harbors alterations in PTEN, ARID1A, and mismatch repair genes and typically presents at earlier stages with more favorable outcomes. Clear cell carcinoma, also linked to endometriosis, is characterized by ARID1A loss and activation of PI3K/AKT signaling and is notable for its relative chemoresistance, whereas mucinous carcinoma is rare, molecularly resembles gastrointestinal malignancies, and similarly responds poorly to standard chemotherapy. This profound biological diversity underscores the need for precision-based therapeutic strategies tailored to tumor-specific molecular features [3,4,5,6,7,8,9].

The incorporation of molecularly targeted therapies into clinical practice has significantly expanded therapeutic options and reshaped the management landscape of ovarian cancer by intervening in key biological pathways that drive tumor growth and survival, including angiogenesis, DNA damage repair, and folate receptor–mediated cellular uptake [11,12,13,14]. Inhibition of vascular endothelial growth factor (VEGF) disrupts tumor angiogenesis and perfusion; poly(ADP-ribose) polymerase (PARP) inhibition exploits homologous recombination deficiency (HRD) to induce synthetic lethality; and folate receptor-alpha (FRα)–targeted strategies enable selective delivery of cytotoxic agents to tumor cells that overexpress this receptor [15,16,17]. Together, these approaches represent a shift away from treatment paradigms based solely on disease stage, tumor burden, or platinum sensitivity toward more individualized, biology-driven therapeutic decision-making.

Although multiple narrative reviews have independently examined antiangiogenic agents, PARP inhibitors, or antibody–drug conjugates in ovarian cancer, fewer have synthesized these modalities within a unified, biomarker-driven clinical framework. The novelty of the present review lies in its integrative analysis of three cornerstone targeted strategies—VEGF inhibition, PARP inhibition, and FRα-directed therapy—across the disease continuum, with particular emphasis on therapeutic sequencing, resistance evolution, and real-world clinical decision-making. By anchoring molecular mechanisms to pivotal trial data and emerging biomarkers, this manuscript aims to clarify how these agents can be optimally deployed, alone or sequentially, to maximize durability of response while anticipating resistance. In doing so, the review provides added value beyond descriptive summaries by offering a forward-looking perspective on treatment integration and future research priorities.

Bevacizumab, a monoclonal antibody targeting VEGF-A, was the first antiangiogenic agent to be widely incorporated into standard ovarian cancer treatment. The pivotal GOG-0218 and ICON7 trials demonstrated that the addition of bevacizumab to platinum–taxane chemotherapy, followed by maintenance therapy, resulted in significant improvements in progression-free survival (PFS), with the greatest benefit observed in patients with high-risk clinical features such as suboptimal cytoreduction or stage IV disease [18,19]. These findings established bevacizumab as a key component of therapy across frontline, recurrent, and platinum-resistant settings.

The subsequent development of PARP inhibitors—including rucaparib, olaparib, and niraparib—further transformed the therapeutic landscape, particularly for patients with BRCA-mutated or HRD-positive tumors [20,21,22].

PARP inhibitors exert their antitumor activity through inhibition of the PARP1/2 enzymes involved in single-strand DNA break repair. In tumors with deficient homologous recombination repair—most notably those harboring BRCA1/2 mutations—PARP inhibition induces synthetic lethality by promoting the accumulation of DNA damage, replication fork collapse, and ultimately cell death [23,24]. In addition to catalytic inhibition, PARP trapping on DNA further enhances cytotoxicity and contributes to clinical efficacy [25].

Several PARP inhibitors have been developed and evaluated in ovarian cancer across frontline and recurrent settings. Olaparib was the first-in-class agent to demonstrate significant benefit as maintenance therapy in BRCA-mutated ovarian cancer and subsequently in broader homologous recombination–deficient populations [26,27]. Its clinical development has included both recurrent platinum-sensitive disease and first-line maintenance following response to platinum-based chemotherapy. Niraparib subsequently demonstrated efficacy as maintenance therapy in newly diagnosed and recurrent ovarian cancer, including in biomarker-selected (HRD-positive) as well as overall study populations [28].

Rucaparib has demonstrated durable and clinically meaningful efficacy in both treatment and maintenance settings, as evidenced by the ARIEL2 and ARIEL3 trials, while maintaining a manageable safety profile [29,30]. Its clinical development program has encompassed recurrent platinum-sensitive ovarian cancer and biomarker-defined subgroups, including patients with HRD, further establishing its role across distinct therapeutic contexts [31]. PARP inhibitors are now foundational therapies in both newly diagnosed and recurrent ovarian cancer, and ongoing clinical investigations are evaluating combination strategies with antiangiogenic agents and immune checkpoint inhibitors (ICIs) to enhance and extend therapeutic benefit.

Although this manuscript discusses rucaparib in greater detail as a representative example in selected sections, the underlying therapeutic principles—including synthetic lethality, HRD as a predictive biomarker, and mechanisms of acquired resistance—are broadly applicable across the PARP inhibitor class [24,32].

More recently, mirvetuximab soravtansine, an FRα-targeted antibody–drug conjugate (ADC), has emerged as a significant therapeutic advance for patients with platinum-resistant ovarian cancer. The SORAYA and MIRASOL trials reported substantial improvements in objective response rates (ORRs), disease control, and tolerability among patients with high FRα expression, representing the first meaningful therapeutic progress for this population in several years [33,34]. By selectively delivering a potent maytansinoid payload to FRα-overexpressing tumor cells, mirvetuximab soravtansine achieves targeted cytotoxicity while minimizing off-target toxicity to normal tissues.

Collectively, VEGF inhibitors, PARP inhibitors, and FRα-targeted ADCs exemplify the ongoing transition toward biomarker-driven, pathway-specific therapy in ovarian cancer [35,36]. Beyond summarizing efficacy data, this review emphasizes how these agents can be strategically positioned across lines of therapy, informed by tumor biology, prior treatment exposure, and evolving resistance mechanisms. Current and future research efforts focus on elucidating mechanisms of acquired resistance, refining predictive biomarkers, and optimizing rational combination regimens, with the overarching goal of extending therapeutic benefit to a broader and more diverse population of patients affected by this disease [37,38].

For this synthesis, a narrative literature review was conducted using the PubMed and Scopus databases. Search terms included “bevacizumab,” “rucaparib,” and “mirvetuximab soravtansine,” in combination with “targeted therapy” and “ovarian cancer.” Peer-reviewed publications published between 2005 and 2025 were selected based on relevance, methodological rigor, and contribution to understanding therapeutic efficacy, mechanisms of action, and resistance. Both preclinical and clinical studies were included when they provided meaningful mechanistic insight or informed clinical decision-making. This approach yielded a comprehensive synthesis of current evidence highlighting the evolving roles, benefits, and limitations of bevacizumab, rucaparib, and mirvetuximab soravtansine in contemporary ovarian cancer management.

**Figure 1:**
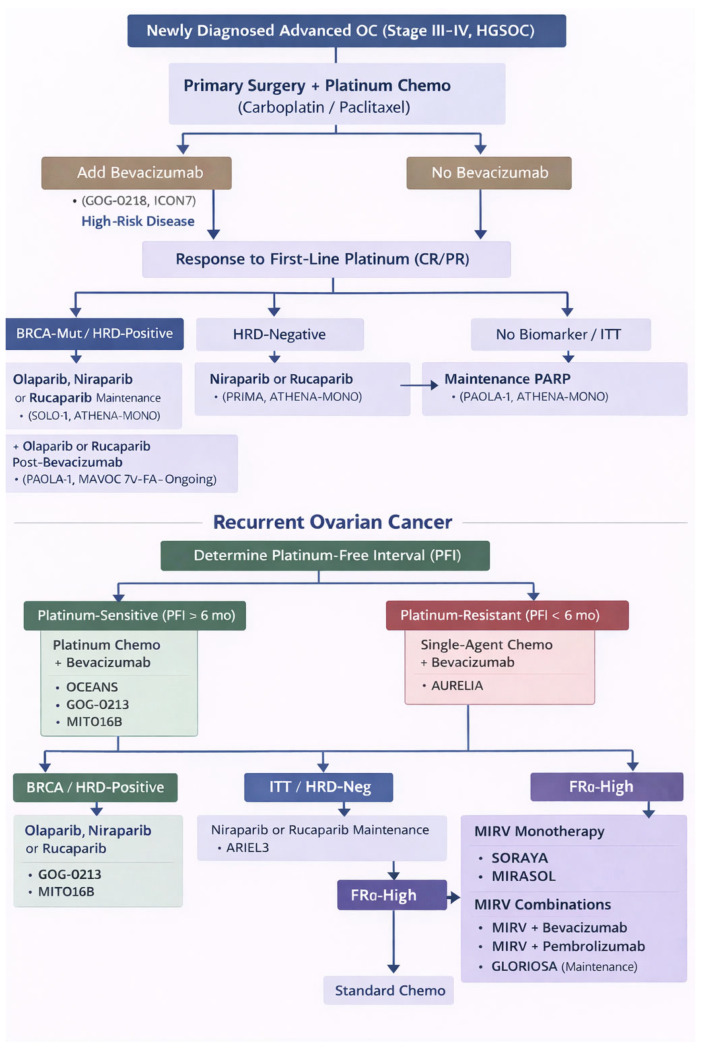
Ovarian cancer clinical decision algorithm with key trial anchors for biomarker-driven therapy, where OC—ovarian cancer; HGSOC—high-grade serous ovarian cancer; CR—complete response; PR—partial response; PFI—platinum-free interval; ITT—intention-to-treat; HRD—homologous recombination deficiency; BRCA—breast cancer gene (BRCA1/2); FRα—folate receptor alpha; PARP—poly(ADP-ribose) polymerase; MIRV—mirvetuximab soravtansine; SOLO-1—study of olaparib in first-line maintenance; PRIMA—niraparib first-line maintenance study; ATHENA-MONO—rucaparib first-line maintenance study; PAOLA-1—olaparib + bevacizumab first-line maintenance study; MAVOC 7V-FA—PARP inhibitor maintenance (e.g., olaparib or rucaparib) after prior bevacizumab study; ARIEL3—rucaparib maintenance in recurrent platinum-sensitive OC; OCEANS—platinum-sensitive recurrent OC trial; GOG-0218—gynecologic oncology group frontline bevacizumab trial; GOG-0213—platinum-sensitive recurrent OC trial; MITO16B—Italian multicenter trial in platinum-sensitive recurrence; ICON7—international collaboration on ovarian neoplasms trial 7; AURELIA—platinum-resistant OC trial; SORAYA—MIRV in FRα-high platinum-resistant OC; MIRASOL—MIRV vs. chemotherapy in FRα-high platinum-resistant OC; GLORIOSA—MIRV maintenance study. For newly diagnosed disease, patients undergo primary surgery followed by platinum-based chemotherapy (carboplatin/paclitaxel) with or without bevacizumab (GOG-0218, ICON7). After response to first-line platinum (CR/PR), maintenance therapy is selected according to biomarker status. Patients with BRCA-mutated/HRD-positive tumors may receive olaparib, niraparib, or rucaparib maintenance (SOLO-1, ATHENA-MONO), with consideration of post-bevacizumab strategies (PAOLA-1—olaparib, MAMOC—rucaparib). HRD-negative patients may receive niraparib or rucaparib maintenance (PRIMA, ATHENA-MONO). In patients without biomarker selection (ITT), maintenance PARP inhibitor therapy is supported by PAOLA-1 and ATHENA-MONO. For recurrent ovarian cancer, management is guided by platinum-free interval (PFI). Platinum-sensitive disease (PFI > 6 months) is treated with platinum-based chemotherapy ± bevacizumab (OCEANS, GOG-0213, MITO16B), followed by biomarker-directed maintenance: PARP inhibitor therapy for BRCA/HRD-positive disease (ARIEL3), niraparib or rucaparib maintenance for ITT/HRD-negative populations (ARIEL3), and MIRV strategies for FRα-high tumors (SORAYA, MIRASOL). Platinum-resistant disease (PFI < 6 months) is treated with single-agent chemotherapy ± bevacizumab (AURELIA), with FRα-high tumors eligible for MIRV monotherapy or combination approaches (including bevacizumab or pembrolizumab; GLORIOSA maintenance). Standard chemotherapy remains an option where appropriate.

Figure 1 illustrates a clinical decision algorithm for ovarian cancer incorporating key trial anchors to guide biomarker-driven therapy.

## 2. Bevacizumab—VEGF Inhibitor

Bevacizumab, commercially known as Avastin, is a recombinant humanized monoclonal antibody targeting vascular endothelial growth factor A (VEGF-A). It was the first monoclonal antibody developed to inhibit tumor angiogenesis through direct neutralization of VEGF-A signaling and is classified as an antiangiogenic biologic agent. Structurally, bevacizumab is an IgG1 antibody engineered by grafting murine complementarity-determining regions derived from the VEGF-binding antibody A.4.6.1 onto a human IgG1 framework, thereby reducing immunogenicity while preserving binding affinity [39,40].

**Figure 2:**
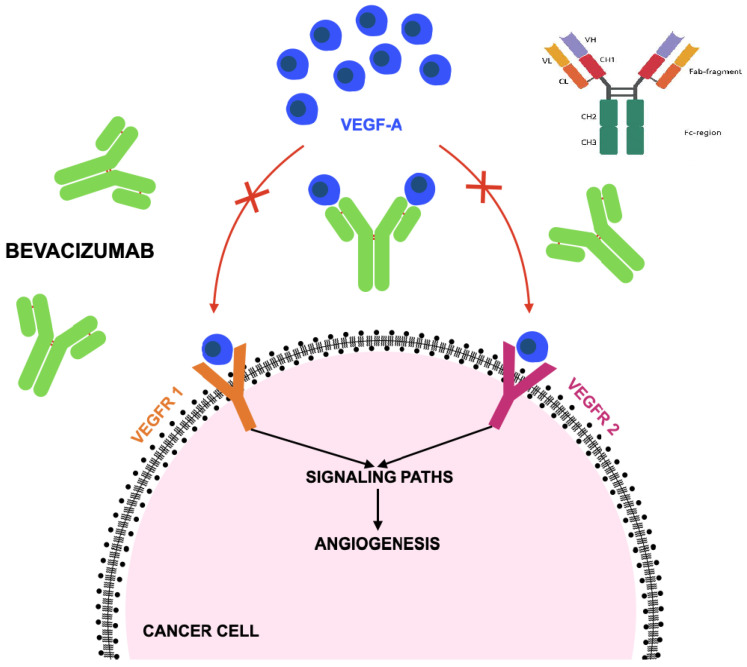
Mechanism of action of bevacizumab according to [45]. Bevacizumab is a full-length IgG1 monoclonal antibody composed of two light and two heavy chains, forming Fab regions for antigen binding and an Fc region for structural support. The drug exerts its anti-tumor effects by binding vascular endothelial growth factor A (VEGF-A) and blocking its interaction with vascular endothelial growth factor receptors (VEGFR-1/2), thereby disrupting angiogenesis, preventing new tumor blood vessel formation, and ultimately suppressing tumor cell growth.

VEGF is a central proangiogenic cytokine that binds VEGF receptors (VEGFR-1 and VEGFR-2) on endothelial cells, activating downstream signaling pathways that promote endothelial cell proliferation, migration, and survival. Bevacizumab binds all biologically active isoforms of VEGF-A, preventing receptor activation and thereby suppressing angiogenesis [41,42]. This inhibition reduces vascular permeability, limits neovascularization, and constrains tumor growth and metastatic spread. A transient phase of “vascular normalization” may occur following treatment initiation, during which immature vessels are pruned and perfusion is temporarily improved, enhancing the delivery of cytotoxic chemotherapy [43]. However, sustained VEGF blockade may lead to excessive vessel regression, exacerbating tumor hypoxia and triggering compensatory activation of alternative proangiogenic pathways such as fibroblast growth factor (FGF) and platelet-derived growth factor (PDGF), thereby contributing to adaptive resistance [44]. The mechanistic basis of bevacizumab’s therapeutic effects is illustrated in Figure 2.

The concept of targeting angiogenesis as a therapeutic strategy was first proposed by Judah Folkman in the 1970s [46]. Subsequent identification of VEGF as a key regulator of tumor vascularization, together with the demonstration that anti-VEGF antibodies inhibited tumor growth in preclinical models, provided the rationale for clinical development. Bevacizumab, developed by Genentech, entered first-in-human testing in 1997. Phase I studies established acceptable safety and tolerability at doses up to 10 mg/kg, with hypertension identified as the principal dose-limiting toxicity [47].

The pivotal phase III AVF2107g trial evaluated bevacizumab in combination with irinotecan, 5-fluorouracil, and leucovorin (IFL) in patients with metastatic colorectal cancer. Compared with chemotherapy alone, bevacizumab significantly improved median OS (20.3 vs. 15.6 months), PFS (10.6 vs. 6.2 months), and ORR (45% vs. 35%) [48]. These results led to U.S. Food and Drug Administration approval in 2004, making bevacizumab the first antiangiogenic agent approved for cancer treatment. Subsequent trials expanded its indications across multiple malignancies.

In ovarian cancer, the GOG-0218 and ICON7 trials established the benefit of incorporating bevacizumab into carboplatin–paclitaxel regimens for advanced disease, resulting in significant prolongation of PFS [18,19]. In the recurrent setting, the OCEANS trial demonstrated that bevacizumab combined with carboplatin–gemcitabine significantly improved PFS in patients with platinum-sensitive recurrent ovarian cancer (12.4 vs. 8.4 months), although no overall survival (OS) benefit was observed, supporting its use at first recurrence [49]. The AURELIA trial further showed that adding bevacizumab to single-agent chemotherapy (weekly paclitaxel, pegylated liposomal doxorubicin, or topotecan) significantly improved PFS (6.7 vs. 3.4 months) and ORRs in platinum-resistant ovarian cancer, supporting its incorporation into treatment strategies [50]. In GOG-0213, bevacizumab combined with paclitaxel–carboplatin at platinum-sensitive recurrence resulted in a significant OS benefit (42.2 vs. 37.3 months), providing the first evidence of an OS advantage for bevacizumab in ovarian cancer [51]. More recently, the MITO16B/ENGOT-ov17 (MaNGO-OV2B) trial demonstrated that continuation of bevacizumab beyond progression, in combination with platinum-based chemotherapy at platinum-sensitive relapse, significantly improved PFS compared with chemotherapy alone, supporting the strategy of bevacizumab rechallenge in appropriately selected patients [52].

Across tumor types, the clinical benefit of bevacizumab is observed more consistently in PFS than in OS, in part due to crossover and the availability of effective post-progression therapies. Meta-analyses have confirmed a statistically significant improvement in PFS across solid tumors, whereas OS benefits remain variable and context-dependent [53,54].

Because VEGF plays a critical role in maintaining normal vascular homeostasis, systemic inhibition is associated with a distinct toxicity profile. Hypertension is among the most common adverse effects, occurring in up to 15% of patients, and is thought to result from reduced nitric oxide production and increased vascular resistance [55]. Proteinuria and renal dysfunction arise from injury to glomerular endothelial cells. Bleeding events—ranging from mild epistaxis to severe gastrointestinal or pulmonary hemorrhage—are relatively common, and arterial thromboembolic events such as myocardial infarction and stroke, while infrequent, occur at increased rates [56,57]. Gastrointestinal perforation, although rare (approximately 2%), is potentially life-threatening and necessitates immediate discontinuation of therapy [58]. Impaired wound healing and wound dehiscence may also occur; consequently, bevacizumab is typically withheld for at least 28 days before and after major surgical procedures [59]. Rare but serious complications include reversible posterior leukoencephalopathy syndrome (RPLS) and thrombotic microangiopathy [60].

Resistance to VEGF-targeted therapy, such as bevacizumab, is predominantly mediated through adaptive angiogenic reprogramming. Tumors may upregulate alternative proangiogenic signaling pathways, including fibroblast growth factors (FGF), platelet-derived growth factor (PDGF), and angiopoietin-2 (ANG2), thereby bypassing VEGF blockade [44,61]. In addition, vascular remodeling characterized by increased pericyte coverage can stabilize tumor vasculature and reduce susceptibility to antiangiogenic therapy [43]. Hypoxia induced by VEGF inhibition may activate HIF-1α–driven transcriptional programs that promote tumor cell survival, metabolic reprogramming, and immune suppression within the tumor microenvironment [62]. These adaptive mechanisms underscore the dynamic tumor–stroma interplay under sustained antiangiogenic pressure.

Resistance to bevacizumab develops through multiple mechanisms, including upregulation of alternative angiogenic signaling pathways (FGF, PDGF, ANGPT), vessel co-option, and hypoxia-driven increases in tumor invasiveness [63,64]. These observations have prompted the development of combination strategies incorporating bevacizumab with chemotherapy, radiotherapy, or molecularly targeted agents. In particular, combinations with ICIs have demonstrated synergistic activity [65].

Beyond immunotherapy-based combinations, accumulating mechanistic evidence supports integrating bevacizumab with PARP inhibitors and FRα-targeted therapies. VEGF inhibition–induced hypoxia may downregulate homologous recombination repair genes and increase genomic instability, thereby enhancing sensitivity to PARP inhibition, consistent with established links between hypoxia and DNA damage repair pathways [66,67,68,69]. This biological interaction provides a strong rationale for combining antiangiogenic therapy with DNA damage–targeting agents and may partially explain the observed clinical activity of such regimens [21,29,30].

Combination approaches may introduce additive or synergistic toxicities. When bevacizumab is combined with PARP inhibitors, overlapping adverse events include hypertension, fatigue, anemia, and gastrointestinal toxicity, reflecting the established safety profiles of both drug classes [55,56,57,58]. Careful blood pressure monitoring, early management of proteinuria, and proactive intervention for hematologic toxicities are therefore essential. In combinations involving mirvetuximab soravtansine, ocular toxicities—such as keratopathy and blurred vision—require prophylactic corticosteroid eye drops, dose modifications, and routine ophthalmologic monitoring, in accordance with safety data from pivotal mirvetuximab soravtansine trials [33,34]. When combined with immune checkpoint inhibitors (ICIs), bevacizumab may further increase the incidence of immune-related adverse events by enhancing immune cell infiltration and activation within the tumor microenvironment, as observed in antiangiogenic–ICI studies [65].

VEGF inhibition also exerts broader effects on the tumor microenvironment by normalizing tumor vasculature, improving drug delivery, and facilitating immune cell infiltration [63,64,65,70]. These changes may enhance the efficacy of FRα-targeted antibody–drug conjugates such as mirvetuximab soravtansine, which depend on efficient intratumoral penetration and receptor-mediated internalization. Mirvetuximab soravtansine delivers a tubulin-disrupting maytansinoid payload that induces mitotic arrest and apoptosis; combining this mechanism with angiogenesis inhibition or modulation of DNA damage response pathways may yield complementary cytotoxic effects [33,34].

Strong mechanistic synergy has also been demonstrated between antiangiogenic therapy and immune checkpoint (IC) inhibition. VEGF suppresses dendritic cell maturation, promotes regulatory T-cell expansion, and contributes to the development of an immunosuppressive tumor microenvironment [71,72,73,74,75]. Bevacizumab counteracts these effects by enhancing T-cell infiltration and antigen presentation, whereas ICIs amplify immune activation by blocking inhibitory signaling pathways. These complementary mechanisms have been evaluated in clinical trials across multiple solid tumors; however, definitive efficacy in ovarian cancer remains to be established [65].

Ongoing research focuses on identifying predictive biomarkers to refine patient selection for bevacizumab therapy. Candidate biomarkers—including baseline VEGF levels, circulating endothelial cells, and various genomic signatures—have been investigated; however, none has yet achieved sufficient validation for routine clinical use [76,77,78,79,80]. Therapeutic drug monitoring is not currently standard practice.

Overall, bevacizumab has fundamentally reshaped cancer therapeutics by validating angiogenesis inhibition as an effective anticancer strategy. Its introduction marked a pivotal advance in oncology, catalyzing the development of subsequent VEGF-targeted therapies and establishing vascular modulation as a core principle of modern cancer treatment. Despite modest OS benefits in certain settings and a distinct toxicity profile, bevacizumab remains a cornerstone antiangiogenic agent, frequently incorporated into rational combination regimens with immunotherapy or other targeted approaches. Future directions include improved biomarker-based patient selection, strategies to overcome resistance, and optimization of combination therapies to fully realize the therapeutic potential of VEGF inhibition. Table 1 summarizes treatment-emergent adverse events (TEAEs) associated with bevacizumab and corresponding management strategies, while Table 2 highlights key pivotal trials in ovarian cancer evaluating its efficacy and safety.

## 3. Rucaparib—PARP Inhibitor

Rucaparib, commercially known as Rubraca, is a small-molecule inhibitor of poly(ADP-ribose) polymerase (PARP) enzymes—primarily PARP-1, PARP-2, and PARP-3—developed for malignancies characterized by defective homologous recombination repair (HRR), including tumors harboring BRCA1 or BRCA2 mutations. It is a potent and selective PARP inhibitor with nanomolar affinity and is formulated as an oral tablet [86,87,88].

**Figure 3:**
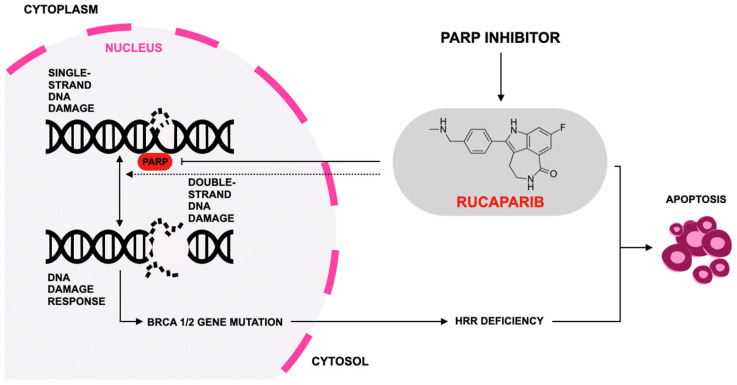
Mechanism of action of rucaparib according to [92]. PARP inhibitors such as rucaparib block the PARP enzyme needed to repair single-strand DNA breaks, causing these lesions to become double-strand breaks during replication that BRCA1/2-mutated, homologous-recombination-deficient (HRD) cells cannot repair using the homologous recombination repair (HRR) pathway, leading to lethal DNA damage accumulation and providing a selective therapeutic strategy that exploits cancer cells’ DNA-repair vulnerabilities.

Rucaparib’s mechanism of action involves inhibition of PARP enzymatic activity, thereby preventing repair of single-strand DNA breaks (SSBs) through the base excision repair pathway. Accumulation of unrepaired SSBs leads to replication fork collapse and formation of double-strand breaks (DSBs). In tumor cells with HRR deficiencies—such as those with pathogenic BRCA1 or BRCA2 mutations—the inability to repair DSBs results in cell death via synthetic lethality [86,89]. In addition to catalytic inhibition, rucaparib traps PARP–DNA complexes on chromatin, generating cytotoxic lesions that impede DNA replication and transcription, thereby amplifying DNA damage and promoting apoptosis [31,90,91]. Figure 3 illustrates the therapeutic mechanism of rucaparib.

The drug’s origins trace to the compound AG014699 (later CO-338 and PF-01367338), which was discovered through structure–activity optimization to achieve potent PARP1 inhibition [93,94]. Early medicinal chemistry studies demonstrated that enhanced PARP trapping and stabilization of DNA–PARP complexes correlated with cytotoxicity in homologous recombination–deficient (HRD) tumor models, establishing the mechanistic basis for clinical development [93,94]. The first-in-human study, initiated in 2003, evaluated intravenous rucaparib in advanced solid tumors and subsequently transitioned to oral dosing, including combination regimens with chemotherapy [95,96]. Pharmacodynamic analyses confirmed effective on-target PARP inhibition, while neutropenia and thrombocytopenia emerged as dose-limiting toxicities attributable to DNA damage accumulation in hematopoietic progenitors [97,98,99].

Subsequent phase I/II trials of oral rucaparib monotherapy in patients with germline BRCA1/2-mutated ovarian carcinoma and other solid tumors established 600 mg twice daily as the recommended phase II dose [86]. These studies defined key pharmacologic properties, including sustained PARP inhibition over the dosing interval and exposure–response relationships consistent with synthetic lethality in HRD tumors. The pivotal Study 10 reported an ORR of 59.3% (95% CI 45.0–72.4%) and a median response duration of 9.7 months in heavily pretreated ovarian cancer patients [100], underscoring the central role of BRCA-dependent DNA repair deficiency in mediating tumor sensitivity to rucaparib.

The ARIEL2 trial extended these findings by stratifying patients with platinum-sensitive relapsed ovarian cancer according to BRCA mutation status and genomic loss of heterozygosity (LOH). Median PFS was 12.8 months in BRCA-mutant tumors, 5.7 months in LOH-high tumors, and 5.2 months in LOH-low tumors, validating LOH as a predictive biomarker for PARP inhibitor response [29]. This biomarker-driven strategy provided one of the earliest clinical demonstrations that HRD signatures beyond BRCA mutations could identify rucaparib-sensitive populations. Phase III ARIEL3 subsequently confirmed rucaparib’s efficacy as maintenance therapy following platinum-based chemotherapy, demonstrating significant PFS prolongation across BRCA-mutated, HRD-positive, and intent-to-treat populations [31]. The confirmatory ARIEL4 trial further demonstrated improved PFS with rucaparib compared with chemotherapy (hazard ratio 0.64) in relapsed BRCA-mutated ovarian carcinoma, completing the evidence base supporting regulatory approval [100].

Building on this foundation, the ATHENA program represents the most comprehensive evaluation of rucaparib in the frontline maintenance setting. ATHENA-MONO, a randomized phase III trial, assessed rucaparib versus placebo as maintenance therapy in patients with newly diagnosed advanced ovarian cancer who achieved a response after first-line platinum-based chemotherapy. By moving rucaparib into the first-line setting, ATHENA-MONO addressed a clinically critical question with greater potential impact than later-line single-arm studies and demonstrated a significant PFS benefit in both HRD tumors and the overall study population [101]. Within the same program, ATHENA-COMBO evaluated whether the addition of the PD-1 inhibitor nivolumab to rucaparib could further improve outcomes in the first-line maintenance setting. Despite a strong biological rationale, this large phase III study showed no PFS advantage for the combination over rucaparib monotherapy, providing important negative evidence to inform clinical practice and future trial design [102].

In parallel, the MAMOC/NOGGO Ov-42 phase III trial addressed a distinct but clinically relevant treatment sequence by enrolling patients who had already received bevacizumab maintenance following first-line carboplatin-based chemotherapy. By comparing rucaparib with placebo—particularly in BRCA-wild-type populations—this study evaluates whether PARP inhibition confers incremental benefit after antiangiogenic therapy [103]. Additionally, early-phase studies investigating rucaparib in combination with bevacizumab established the feasibility, safety profile, and recommended dosing of this doublet. Although not designed to demonstrate definitive efficacy, these trials provided critical translational and clinical support for PARP–antiangiogenic combination strategies and informed the design of subsequent randomized studies [104].

The most frequent TEAEs with rucaparib include nausea, vomiting, fatigue, anemia, and elevated transaminases. These effects reflect both on-target accumulation of unrepaired DNA lesions and off-target mitochondrial and metabolic stress. In pooled analyses of more than 500 patients, grade ≥3 adverse events included anemia (22–28%), fatigue (15–18%), and elevated AST/ALT (10–12%), resulting in dose reductions in nearly half of patients and treatment discontinuation in approximately 17% [31,86]. Myelosuppression—particularly neutropenia and thrombocytopenia—was dose-limiting when combined with chemotherapy, consistent with cumulative DNA damage–mediated marrow suppression [93,97].

Safety considerations are increasingly important as rucaparib is investigated in combination with antiangiogenic agents, ICIs, and other DNA damage response (DDR)–targeting therapies [31,93,97]. In combinations with bevacizumab, overlapping toxicities include hypertension, fatigue, anemia, and gastrointestinal adverse events due to additive VEGF- and PARP-mediated effects [31,86]. Clinical management requires vigilant blood pressure monitoring, early intervention for proteinuria, and proactive treatment of cytopenias [55,56,57,58]. Combinations with ICIs aim to enhance immunogenic cell death but may increase the risk of immune-related colitis, hepatitis, dermatitis, and endocrinopathies [65,70]. DDR-targeting combinations, such as rucaparib with ATR or WEE1 inhibitors, carry a heightened risk of myelosuppression and necessitate individualized dose modifications, underscoring the importance of balancing efficacy with tolerability [31,86,93,97].

Clinical response to rucaparib strongly correlates with HRD biomarkers. The highest response rates are observed in BRCA-mutated and HRD-positive tumors identified via LOH-based assays [105,106,107]. The U.S. Food and Drug Administration–approved companion diagnostic, FoundationFocus™ CDx_BRCA, identifies germline and somatic BRCA1/2 mutations to guide patient selection. Current combination strategies under investigation include rucaparib with bevacizumab or ICIs, aiming to exploit hypoxia-driven HRD, enhance immunogenicity, and overcome intrinsic or acquired resistance [108,109,110].

Resistance to PARP inhibitors frequently involves restoration of homologous recombination (HR) repair capacity. Secondary BRCA1/2 reversion mutations can restore protein function and reverse synthetic lethality [111,112]. Additional mechanisms include replication fork stabilization, loss of proteins regulating DNA repair pathway choice, and epigenetic reactivation of HR-associated genes [24]. Upregulation of drug efflux transporters, such as ABCB1, may further reduce intracellular PARP inhibitor concentrations and contribute to clinical resistance [113]. Collectively, these pathways highlight the genomic plasticity of ovarian cancer under sustained DNA damage response inhibition.

Resistance mechanisms include BRCA reversion mutations restoring HRR function, upregulation of drug efflux transporters, and stabilization of stalled replication forks, which reduce PARP trapping and sensitivity to DNA damage–induced cytotoxicity. Ongoing research aims to overcome these mechanisms through rational combination strategies and more refined predictive biomarkers, including targeting ATR, CHK1, or WEE1 to destabilize fork protection or suppress HRR restoration [114,115,116,117,118,119].

Rucaparib is a potent oral PARP inhibitor with established efficacy in BRCA-mutated and HRD-positive ovarian and prostate cancers. Its development—from preclinical discovery to phase III validation—illustrates the successful clinical translation of synthetic lethality in targeted oncology. Current trials continue to explore its use across additional malignancies, earlier lines of therapy, and synergistic combinations, while long-term safety and mechanisms of resistance remain active areas of investigation [120,121,122,123,124,125,126]. Table 3 summarizes TEAEs and management strategies for rucaparib, and Table 4 outlines key clinical trials in ovarian cancer evaluating its efficacy and safety.

## 4. Mirvetuximab Soravtansine—FRα Pathway Inhibitor

Mirvetuximab soravtansine (MIRV; brand name Elahere) is a first-in-class antibody–drug conjugate (ADC) targeting the FRα pathway. The ADC comprises a humanized IgG1 monoclonal antibody specific for FRα, linked via a cleavable sulfo-SPDB linker to the cytotoxic maytansinoid DM4 payload, with an average drug–antibody ratio (DAR) of approximately 3.5 [129,130]. Owing to its macromolecular composition and heterogeneity, a single molecular formula cannot be assigned to the entire ADC [131,132,133].

**Figure 4:**
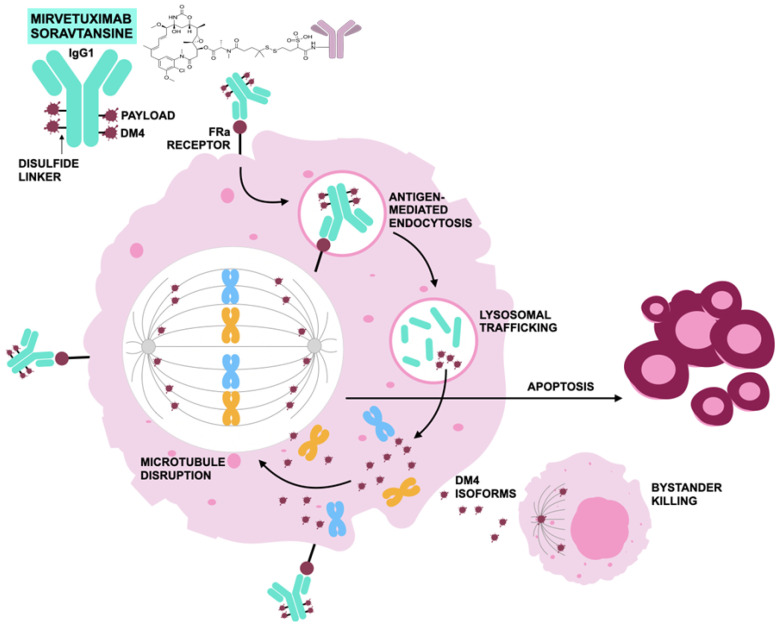
Mechanism of action of mirvetuximab soravtansine according to [143]; mirvetuximab soravtansine binds with high affinity to folate receptor-α on tumor cells, is internalized and degraded in lysosomes to release three active forms of the cytotoxic payload: DM4 (N2′-deacetyl-N2′-(3-mercapto-1-oxopropyl)-maytansine) and its two metabolites—lysine-Nε-sulfo-SPDB-DM4 and S-methyl-DM4, that inhibit tubulin polymerization and induce G2–M (cell cycle transition from the G2 phase as the period before mitosis to the M phase—mitosis) arrest and apoptosis, with the membrane-diffusible S-methyl-DM4 also producing a bystander killing effect.

Mirvetuximab soravtansine selectively binds FRα, a glycosylphosphatidylinositol-anchored cell-surface protein involved in folate transport that is highly expressed in ovarian, endometrial, and other epithelial malignancies, with limited expression in normal tissues [131,134,135]. Upon binding FRα, the ADC–receptor complex undergoes endocytosis, followed by lysosomal cleavage of the linker and intracellular release of DM4 [136,137,138]. DM4 inhibits microtubule polymerization, induces G2/M mitotic arrest, and activates apoptotic pathways. The hydrophobic payload and its membrane-permeable metabolites are thought to contribute to a bystander effect, permitting cytotoxic activity in adjacent tumor cells with low or absent FRα expression [135,139,140,141,142]. Figure 4 illustrates the mechanism of action of mirvetuximab soravtansine.

The first clinical evaluation of mirvetuximab soravtansine occurred in an open-label phase I dose-escalation trial in patients with FRα-positive, platinum-resistant epithelial ovarian cancer (EOC). This study established a recommended phase II dose of 6 mg/kg based on adjusted ideal body weight and demonstrated early clinical activity with a manageable safety profile [33].

The pivotal randomized phase III FORWARD I trial (NCT02631876) compared mirvetuximab soravtansine monotherapy with investigator’s-choice chemotherapy in patients with platinum-resistant ovarian cancer and medium or high FRα expression. Although the primary endpoint of PFS was not met in the intent-to-treat population (HR 0.98; *p* = 0.897), a clinically meaningful improvement in median PFS was observed in the FRα-high subgroup (6.7 vs. 3.9 months), underscoring the importance of biomarker-driven patient selection [144]. Building on these findings, the single-arm phase II SORAYA trial (NCT04296890) enrolled patients with FRα-high, platinum-resistant ovarian cancer who had received 1–3 prior therapies, including bevacizumab, and met its primary endpoint with an ORR of 32.4% and a median duration of response (DOR) of 6.9 months, supporting accelerated U.S. Food and Drug Administration approval in November 2022 [33,131]. Confirmatory evidence was subsequently provided by the phase III MIRASOL trial (NCT04209855), which demonstrated significant improvements in PFS (5.62 vs. 3.98 months; *p* < 0.001), ORR (42.3% vs. 15.9%; *p* < 0.001), and OS (16.46 vs. 12.75 months; HR 0.67; *p* = 0.005) compared with chemotherapy, leading to full U.S. Food and Drug Administration approval in 2024 and European Commission approval in 2025 [34,131,145,146]. Across studies, pooled analyses have demonstrated consistent efficacy in FRα-high tumors, with an ORR of approximately 36% and a median PFS of approximately 6.7 months [147].

In parallel with the development of mirvetuximab soravtansine monotherapy, the multicenter phase Ib/II FORWARD II trial explored the safety and preliminary efficacy of mirvetuximab soravtansine in combination with bevacizumab, pembrolizumab, or carboplatin in patients with FRα-positive recurrent ovarian cancer across both platinum-resistant and platinum-sensitive settings. These combination cohorts demonstrated manageable toxicity profiles and early signals of enhanced antitumor activity compared with mirvetuximab soravtansine monotherapy, particularly for the mirvetuximab soravtansine–bevacizumab regimen, thereby providing a strong biological and clinical rationale for combining FRα-targeted antibody–drug conjugates with antiangiogenic or immunotherapeutic agents [148,149]. Building on these data, the ongoing phase III GLORIOSA trial is evaluating mirvetuximab soravtansine plus bevacizumab versus bevacizumab alone as maintenance therapy in patients with FRα-high ovarian cancer who have responded to second-line platinum-based chemotherapy combined with bevacizumab. By addressing a common real-world treatment sequence and moving mirvetuximab soravtansine earlier in the disease course, GLORIOSA represents a critical step in defining the long-term role of FRα-targeted therapy within ovarian cancer treatment algorithms [150].

Combination strategies—including mirvetuximab soravtansine with bevacizumab, ICIs, and platinum-based chemotherapy—are under active investigation and show early evidence of synergistic activity. Mechanistically, antiangiogenic therapy normalizes tumor vasculature, improving ADC delivery and intratumoral penetration, while ICIs may amplify the immunogenic effects of DM4-induced tumor cell death. Platinum-based chemotherapy may induce DNA damage that sensitizes tumor cells to mirvetuximab soravtansine–mediated cytotoxicity, collectively producing complementary antitumor effects. These combination regimens, however, may introduce additive or synergistic toxicities requiring careful management. For example, ocular toxicity associated with mirvetuximab soravtansine may be exacerbated in combinations with agents that induce microvascular or inflammatory stress, while overlapping hematologic or gastrointestinal adverse events may occur with chemotherapy or antiangiogenic therapy. Proactive management strategies include ophthalmologic monitoring, dose adjustments, prophylactic ocular lubrication, and close surveillance for hematologic or gastrointestinal adverse events [147,151,152].

Resistance to FRα-targeted antibody–drug conjugates (ADCs), such as mirvetuximab soravtansine, may arise through both target-dependent and payload-dependent mechanisms. Heterogeneous or reduced FRα expression can limit effective antibody binding and internalization [153]. Alterations in receptor-mediated endocytosis and intracellular trafficking may impair lysosomal processing and cytotoxic payload release [154]. Additionally, resistance to the microtubule-targeting payload may develop through tubulin alterations or increased expression of multidrug resistance transporters, resulting in efflux pump–mediated drug export [155]. Collectively, these mechanisms underscore the importance of sustained antigen expression and intact intracellular processing for optimal ADC efficacy.

Common adverse events with mirvetuximab soravtansine monotherapy include blurred vision (≈41–43%), keratopathy (≈30%), nausea (≈41%), diarrhea (≈39%), and fatigue (≈35%). Grade ≥3 toxicities (48%) are primarily ocular events and fatigue, although discontinuation rates remain relatively low (approximately 12%) [33,132]. Ocular toxicity is attributed to DM4-mediated microtubule disruption in corneal epithelial cells and is managed with dose modifications, prophylactic ocular lubrication, and routine ophthalmologic monitoring [147,151].

High FRα expression—defined as ≥75% of tumor cells demonstrating ≥2+ membrane staining using the VENTANA FOLR1 RxDx assay—is essential for optimal patient selection and therapeutic efficacy [147,156,157]. Collectively, these characteristics position mirvetuximab soravtansine as a paradigm of precision ADC therapy in ovarian cancer, combining targeted receptor engagement with potent intracellular cytotoxicity. Its pharmacologic profile and emerging combination strategies provide clinically meaningful benefit in a population with limited treatment options while underscoring the importance of monitoring and managing additive toxicities [158,159,160,161]. Table 5 summarizes treatment-emergent adverse events and management strategies for mirvetuximab soravtansine, while Table 6 highlights major clinical trials in ovarian cancer evaluating its efficacy and safety, including completed, ongoing, and planned studies.

## 5. Prospective Developments and Implications for Clinical Practice

The therapeutic landscape of ovarian cancer has been fundamentally transformed by the advent of targeted therapies directed against the VEGF, PARP, and FRα pathways. These approaches have redefined modern treatment algorithms by enabling biomarker-driven patient selection, tailoring therapy to molecular vulnerabilities, and exploiting tumor-specific dependencies. Their integration marks a shift from traditional cytotoxic regimens toward precision oncology frameworks that account for intratumoral heterogeneity, dynamic molecular evolution, and tumor microenvironmental influences as key determinants of therapeutic efficacy. However, despite these advances, the clinical impact of targeted therapies remains constrained by several important limitations that warrant critical consideration.

Key challenges include intrinsic and acquired resistance, variability in target expression both between and within patients, and unresolved questions regarding the optimal sequencing, duration, and combination of therapies. In addition, treatment-related toxicities, while often more manageable than those associated with conventional cytotoxic chemotherapy, remain clinically meaningful and may limit long-term adherence—particularly in the context of chronic maintenance strategies. Accessibility and cost represent additional barriers, as the widespread implementation of biomarker testing, antibody–drug conjugates, and prolonged targeted therapy may exacerbate disparities across health systems and geographic regions. These considerations underscore the need for treatment strategies that not only improve efficacy but also preserve tolerability, affordability, and real-world feasibility.

Emerging strategies—including deubiquitinase (DUB) inhibitors, nanoparticle-based delivery systems, and bispecific antibody platforms—reflect ongoing innovation and highlight the necessity of integrated approaches that address both tumor biology and the broader clinical context [164,165,166,167,168,169]. Future directions are expected to emphasize refined biomarker-guided allocation, mechanistically rational combination strategies, and advanced delivery technologies designed to maximize therapeutic index while minimizing systemic toxicity and financial burden [170,171,172,173].

The integration of biomarker testing into routine clinical workflows is essential for optimizing therapeutic sequencing in epithelial ovarian cancer. With the expanding use of PARP inhibitors and antibody–drug conjugates, timely and appropriate assessment of BRCA mutation status, HRD, and FRα expression has become central to individualized treatment planning [174,175].

BRCA1/2 testing is recommended at the time of diagnosis for all patients with epithelial ovarian cancer, regardless of family history [174]. Germline testing should be performed first because of its therapeutic and hereditary implications. If germline testing is negative, somatic tumor testing is recommended to identify acquired BRCA mutations [175]. Identification of pathogenic BRCA variants directly informs eligibility for PARP inhibitor therapy in both first-line maintenance and recurrent settings, given the established benefit of PARP inhibition in BRCA-mutated disease [26,27]. Early testing ensures that maintenance strategies can be implemented without delay following response to platinum-based chemotherapy.

HRD testing is typically performed on tumor tissue at diagnosis or during first-line treatment planning. Commercial genomic instability assays evaluate loss of heterozygosity, telomeric allelic imbalance, and large-scale state transitions as markers of genomic scarring [176]. HRD status has predictive value for PARP inhibitor benefit beyond BRCA-mutated tumors, particularly in the maintenance setting [27,28]. However, important limitations remain, including inter-assay variability, evolving definitions of HRD positivity, and the dynamic nature of homologous recombination restoration over time [32]. In recurrent disease, prior PARP inhibitor exposure may lead to reversion mutations that restore homologous recombination function, thereby complicating biomarker interpretation.

FRα expression testing is required before treatment with FRα-targeted antibody–drug conjugates such as mirvetuximab soravtansine. Assessment is performed using immunohistochemistry, with expression thresholds defined according to criteria used in pivotal clinical trials [153]. In the platinum-resistant setting, high FRα expression has been associated with improved clinical benefit from mirvetuximab soravtansine compared with chemotherapy [143,153,177]. Practical considerations include intratumoral heterogeneity, potential discrepancies between archival and recent biopsy specimens, and modulation of antigen expression following prior systemic therapy.

Within the domain of VEGF inhibition, bevacizumab remains the benchmark antiangiogenic agent for ovarian cancer, consistently improving PFS across first-line, interval debulking, and recurrent disease settings [18,178]. However, the magnitude and durability of benefit are variable, and OS gains have been inconsistent, raising questions regarding optimal patient selection and treatment duration. Resistance frequently emerges through upregulation of alternative proangiogenic pathways, extracellular matrix remodeling, pericyte-mediated vascular stabilization, and hypoxia-driven metabolic adaptation, collectively diminishing long-term efficacy. Moreover, bevacizumab-associated toxicities—including hypertension, proteinuria, thromboembolic events, and gastrointestinal perforation—necessitate careful patient selection and vigilant monitoring. To address these limitations, contemporary trials are evaluating combinations of bevacizumab with ICIs and PARP inhibitors, leveraging its capacity to normalize tumor vasculature, reduce hypoxia, enhance immune infiltration, and improve drug delivery. This strategy positions antiangiogenic therapy as a microenvironment-modifying backbone capable of potentiating immunotherapy and DNA damage response (DDR)–targeted approaches, although the optimal sequencing and safety of such combinations remain active areas of investigation [179,180,181,182].

PARP inhibitors, including rucaparib, have revolutionized the management of high-grade serous ovarian carcinoma by exploiting defects in homologous recombination DNA repair, particularly in BRCA1/2-mutated and HRD-positive tumors in which synthetic lethality yields robust clinical responses [183,184]. Nonetheless, their long-term utility is limited by the emergence of resistance mechanisms, including BRCA reversion mutations restoring homologous recombination proficiency, upregulation of drug efflux transporters, stabilization of stalled replication forks, and DDR pathway rewiring that circumvents PARP dependence [185,186]. In addition, cumulative toxicities—such as anemia, thrombocytopenia, fatigue, and rare cases of myelodysplastic syndrome—pose challenges for prolonged maintenance therapy, particularly in older or comorbid populations. Strategies to overcome resistance include combination regimens targeting complementary DDR pathways (ATR, WEE1, CHK1), integration with ICIs, and pairing with antiangiogenic agents to exploit immunologic and microenvironmental vulnerabilities [187,188,189]. Outstanding clinical questions include the role of PARP inhibitor rechallenge, optimal management after prior PARP exposure, and expansion of benefit to HRD-intermediate or homologous recombination–proficient tumors. Advances in circulating tumor DNA (ctDNA) profiling, methylation-based biomarkers, and liquid biopsy–based monitoring may enable real-time detection of emerging resistance and inform adaptive, personalized treatment strategies [190,191].

FRα-targeted therapy, exemplified by mirvetuximab soravtansine, represents a major advance in precision oncology by selectively targeting a glycoprotein highly expressed in epithelial ovarian cancers while sparing most normal tissues [192,193,194]. The pivotal SORAYA and MIRASOL trials demonstrated superior response rates, improved tolerability, and favorable patient-reported outcomes in FRα-high, platinum-resistant disease compared with standard chemotherapy, establishing mirvetuximab soravtansine as a key therapeutic option in this historically refractory population [34]. Nevertheless, clinical implementation raises unresolved questions regarding optimal FRα expression thresholds, resistance mechanisms, sequencing after prior ADC exposure, and access to standardized, reproducible testing platforms. Ongoing trials, including GLORIOSA and PICCOLO, aim to define its role earlier in the disease course and in combination or maintenance settings to improve response durability [153]. Future development efforts include next-generation FRα-targeted ADCs with optimized payloads, enhanced linker stability, improved internalization, and bispecific constructs capable of engaging immune effector cells. Rational combinations with ICIs or DNA-damaging agents may further enhance efficacy, although safety, cost, and cumulative toxicity will require careful evaluation [195,196]. Artificial intelligence–assisted imaging, digital pathology, and transcriptomic profiling may improve the accuracy and reproducibility of FRα assessment, thereby optimizing patient selection.

Although this review summarizes pivotal clinical trial outcomes across antiangiogenic agents, PARP inhibitors, and FRα-targeted ADCs, careful methodological interpretation is warranted. Cross-trial comparisons are inherently limited because of substantial heterogeneity in study design and patient populations. Trials differ with respect to platinum sensitivity (platinum-sensitive versus platinum-resistant cohorts), number of prior treatment lines, maintenance versus active treatment settings, and biomarker enrichment strategies (e.g., BRCA-mutated, HRD-positive, or FRα-high populations) [197,198]. In addition, variability in HRD assays and companion diagnostics introduces further complexity when comparing outcomes across studies [176]. Importantly, evolving standards of care—including prior exposure to PARP inhibitors or bevacizumab—also influence trial results and limit historical comparability.

Accordingly, indirect comparisons across antiangiogenic trials, PARP inhibitor maintenance studies, and FRα-targeted ADC trials should be interpreted with caution. Differences in eligibility criteria, stratification factors, control arms, and post-progression treatments may substantially affect reported efficacy outcomes [197]. Apparent variations in median PFS or ORRs across studies should not be construed as evidence of relative superiority in the absence of head-to-head randomized data.

Interpretation of clinical endpoints also requires nuance. While PFS improvement is a commonly used primary endpoint—particularly in maintenance settings—it does not consistently translate into OS benefit [199]. In several first-line maintenance trials, OS data remain immature at the time of reporting, limiting definitive conclusions regarding long-term survival impact [27,28]. Furthermore, crossover designs and access to effective subsequent therapies may attenuate OS differences between treatment arms, thereby complicating survival analyses [199]. Variability in follow-up duration, adverse event reporting standards, and definitions of treatment discontinuation further challenges cross-study safety comparisons.

By incorporating these considerations, this review promotes balanced interpretation of the evidence base, minimizes the risk of overgeneralization, and strengthens the academic rigor of the discussion surrounding contemporary therapeutic strategies in ovarian cancer.

The convergence of VEGF inhibition, PARP inhibition, and FRα-directed cytotoxic delivery provides a compelling framework for rational triplet strategies that simultaneously disrupt angiogenesis, DNA repair, and receptor-mediated drug delivery. Preclinical studies suggest synergistic interactions: VEGF blockade enhances immune infiltration and drug access through vascular normalization; PARP inhibition promotes immunogenic cell death and interferon signaling; and FRα-targeted ADC cytotoxicity may be amplified in immune-active or vascularly normalized tumors [200,201]. However, translating these strategies into clinical practice will require careful attention to overlapping toxicities, optimal sequencing, patient selection, and cost-effectiveness, as well as validation in biomarker-driven trials.

Innovations in ADC engineering—including site-specific conjugation, advanced linker chemistries, and next-generation payloads with improved safety profiles—are expanding the therapeutic window of FRα-targeted agents. Similarly, next-generation PARP inhibitors with enhanced catalytic and trapping activity may help overcome resistance while preserving tolerability [202,203]. Incorporation of real-world evidence, patient-reported outcomes, and adaptive clinical trial designs will be critical to defining long-term benefit–risk profiles and ensuring equitable access across diverse patient populations.

Collectively, the integration of VEGF, PARP, and FRα inhibition exemplifies biologically rational, precision-guided therapy in gynecologic malignancies. Sustained progress will depend on multidisciplinary collaboration across molecular biology, pharmacology, immunology, data science, and clinical oncology to address unresolved clinical questions, mitigate toxicity, improve affordability, and translate mechanistic insights into durable and equitable clinical benefit [204,205,206].

As oncologic outcomes improve, increasing numbers of long-term survivors necessitate greater attention to survivorship issues, including quality of life and fertility preservation. This consideration is particularly relevant for reproductive-age women with ovarian or endometrial cancer, for whom oocyte vitrification and other fertility-preserving strategies should be discussed early in treatment planning [207]. Contemporary management increasingly incorporates neoadjuvant chemotherapy, targeted therapies, and minimally invasive surgical approaches. Bevacizumab-based regimens [208] and neoadjuvant chemotherapy [209] have demonstrated efficacy and acceptable safety, potentially allowing greater flexibility for fertility preservation; however, gonadotoxic risks persist and mandate timely counseling [207].

Emerging insights into the epigenetic landscape of endometrial cancer—including chromatin modifications—have enhanced understanding of tumor biology and therapeutic response [210]. These advances may inform personalized treatment, reproductive counseling, and long-term surveillance strategies. Quality-of-life outcomes following extensive surgical procedures, such as pelvic exenteration, further underscore the importance of addressing the physical, psychological, and reproductive consequences of treatment [211]. Fertility preservation therefore represents a critical component of survivorship care and patient-centered oncology [207].

Minimally invasive surgical approaches, including conventional and minilaparoscopic hysterectomy, demonstrate favorable perioperative outcomes and reduced morbidity, potentially facilitating fertility-sparing options while decreasing treatment burden. These approaches also raise medico-legal considerations, reinforcing the importance of informed consent and adherence to evidence-based guidelines [212]. The growing demand for fertility preservation in oncology carries ethical and legal implications, and failure to address reproductive risks and options may adversely affect patient satisfaction and long-term psychosocial outcomes [207]. Integrating fertility preservation into standard oncologic care is thus both a clinical necessity and an ethical imperative.

**Figure 5:**
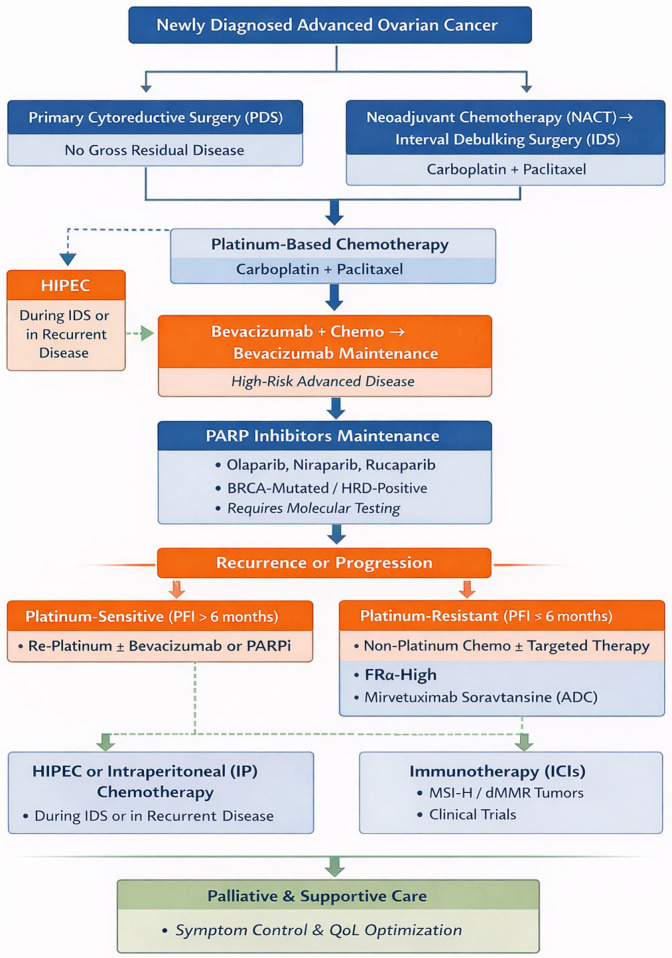
Contemporary management of ovarian cancer, where PDS—primary debulking surgery (primary cytoreductive surgery); NACT—neoadjuvant chemotherapy; IDS—interval debulking surgery; HIPEC—hyperthermic intraperitoneal chemotherapy; IP—intraperitoneal (chemotherapy); PARP—poly (ADP-ribose) polymerase; PARPi—PARP inhibitor; BRCA—breast cancer gene (BRCA1/BRCA2); HRD—homologous recombination deficiency; PFI—platinum-free interval; FRα—folate receptor alpha; ADC—antibody–drug conjugate; ICI—immune checkpoint inhibitor; MSI-H—microsatellite instability–high; dMMR—deficient mismatch repair; and QoL—quality of life. The figure illustrates the contemporary management pathway for newly diagnosed advanced ovarian cancer beginning with either PDS, aiming for no gross residual disease, or NACT followed by IDS using carboplatin plus paclitaxel. All patients subsequently receive platinum-based chemotherapy (carboplatin and paclitaxel). In selected patients with high-risk advanced disease, bevacizumab may be administered concurrently with chemotherapy and continued as maintenance therapy. Following response to platinum treatment, maintenance therapy with a PARP inhibitor (olaparib, niraparib, or rucaparib) may be offered in biomarker-selected populations, particularly those with BRCA mutations or HRD, emphasizing the need for molecular testing. At recurrence or progression, management is stratified according to the PFI. Patients with platinum-sensitive disease (PFI > 6 months) may receive platinum re-treatment with or without bevacizumab or a PARP inhibitor, whereas patients with platinum-resistant disease (PFI ≤ 6 months) are typically treated with non-platinum chemotherapy with or without targeted therapy. In tumors demonstrating high FRα expression, mirvetuximab soravtansine may be considered. The algorithm also incorporates additional therapeutic strategies. HIPEC may be administered during interval debulking surgery in the frontline setting or in selected recurrent cases. Immune checkpoint inhibitors may be considered in biomarker-selected populations, particularly in tumors exhibiting MSI-H or dMMR, and within clinical trial settings, including select frontline scenarios. Across all stages of disease, palliative and supportive care—including symptom control and quality-of-life optimization—remains an essential and continuous component of patient management.

Table 7 presents a clinically focused overview of current treatment strategies for epithelial ovarian cancer, outlining indications, representative regimens or agents, key biomarkers and toxicity considerations, and supporting guideline and clinical trial evidence. Figure 5 illustrates the contemporary management pathway of ovarian cancer.

## 6. Conclusions

The advent of targeted therapies against the VEGF, PARP, and FRα pathways has fundamentally transformed the therapeutic landscape of epithelial ovarian cancer. Bevacizumab, rucaparib, and mirvetuximab soravtansine exemplify three complementary strategies that address tumor angiogenesis, genomic instability, and receptor-mediated cytotoxicity. Their integration into clinical practice has improved outcomes in selected patient populations and underscores the central role of precision medicine in gynecologic oncology.

VEGF inhibition with bevacizumab provided early proof that modulation of the tumor microenvironment can confer meaningful clinical benefit. PARP inhibitors subsequently revolutionized care by exploiting synthetic lethality in BRCA-mutated and homologous recombination–deficient tumors, establishing durable maintenance strategies in both frontline and recurrent settings. FRα-targeted antibody–drug conjugates, such as mirvetuximab soravtansine, have extended precision therapy into the platinum-resistant setting, offering a critical therapeutic advance for patients previously limited to palliative chemotherapy. Collectively, these developments reflect the evolution of ovarian cancer treatment into a biologically informed discipline in which tumor-specific molecular characteristics increasingly guide therapeutic selection.

Despite these advances, substantial challenges remain. Therapeutic resistance, optimal sequencing, cumulative toxicity, and economic considerations continue to limit long-term benefit. The development of robust, clinically actionable biomarkers—such as dynamic measures of FRα expression, refined assessments of homologous recombination repair competency, and angiogenic signatures—will be essential to optimize patient selection and guide rational combination strategies. Future progress will likely depend on synergistic approaches incorporating ICIs and next-generation DNA damage response modulators to enhance antitumor immunity and circumvent mechanisms of resistance.

Targeting VEGF, PARP, and FRα represents a decisive shift from empiric cytotoxic therapy toward mechanism-based, individualized management of ovarian cancer. Continued translational research, innovative clinical trial design, and multidisciplinary collaboration will be critical to defining the optimal integration of these agents within multimodal treatment paradigms. Ultimately, the goal remains to transform ovarian cancer into a chronic, manageable disease through earlier, more strategic intervention and precise molecular targeting.

## Figures and Tables

**Table 1:** TEAEs and management strategies for bevacizumab according to [81,82,83,84,85], where TEAE—treatment-emergent adverse event; VEGF—vascular endothelial growth factor; BP—blood pressure; ACE—angiotensin-converting enzyme; CCB—calcium channel blocker; GI—gastrointestinal; MI—myocardial infarction; DVT—deep vein thrombosis; PE—pulmonary embolism.

TEAE	Frequency/Severity	Timing/ Clinical Features	Recommended Management
Hypertension	Common; usually grade 1–2, occasionally grade ≥3	Typically develops within weeks of initiation; often asymptomatic; sustained BP elevation	Monitor BP before each cycle and at home; initiate antihypertensive therapy (ACE inhibitors or calcium channel blockers preferred); withhold bevacizumab for severe or uncontrolled hypertension until controlled.
Proteinuria	Common; mostly low grade, rare nephrotic syndrome	Often cumulative; detected on routine urinalysis; may be asymptomatic	Routine urinalysis or protein/creatinine ratio; confirm ≥2+ proteinuria with 24 h urine collection; hold therapy for nephrotic-range proteinuria; consider nephrology referral.
Hemorrhage (e.g., epistaxis, GI bleeding)	Minor bleeding common; major bleeding uncommon but serious	Mucocutaneous bleeding most frequent; GI or intracranial hemorrhage rare	Clinical surveillance for bleeding symptoms; supportive care for minor events; permanently discontinue bevacizumab for severe or life-threatening hemorrhage.
Gastrointestinal Perforation	Rare (<2%); potentially fatal	Acute abdominal pain, fever, peritonitis; often associated with bowel involvement or inflammation	Immediate discontinuation of bevacizumab; urgent surgical evaluation and supportive management required.
Delayed Wound Healing	Uncommon; increased perioperatively	Impaired healing or wound dehiscence following surgery	Withhold bevacizumab ≥28 days before and after major surgery; resume only after complete wound healing is confirmed.
Arterial Thromboembolic Events (e.g., MI, stroke)	Uncommon; higher risk in elderly patients and those with vascular comorbidities	Acute neurologic deficits or cardiac ischemic symptoms	Discontinue bevacizumab following major arterial events; manage according to standard cardiovascular or neurologic guidelines.
Venous Thromboembolism (e.g., DVT, PE)	Common in oncology populations; causality multifactorial	Leg swelling, chest pain, dyspnea; may occur at any treatment phase	Initiate anticoagulation per clinical guidelines; continuation of bevacizumab should be individualized based on risk–benefit assessment.
Infusion-Related Reactions	Uncommon; usually mild to moderate (grade 1–2), severe reactions rare	Typically occur during or shortly after infusion; symptoms may include fever, chills, flushing, headache, hypertension, or hypersensitivity reactions	Monitor patients during and after infusion; manage mild reactions with infusion interruption, antihistamines, and/or antipyretics; resume at a slower infusion rate if symptoms resolve; permanently discontinue bevacizumab for severe or life-threatening reactions (e.g., anaphylaxis).

**Table 2:** Major pivotal clinical trials of bevacizumab, where PFS—progression-free survival; OS—overall survival; FIGO—International Federation of Gynecology and Obstetrics; GOG—Gynecologic Oncology Group; PS—performance status; GI—gastrointestinal; ECOG—Eastern Cooperative Oncology Group; ORR—objective response rate; and PLD—pegylated liposomal doxorubicin.

Trial	Population/ Cancer Setting	Design/ Combination	Key Findings	Inclusion/ Eligibility Criteria
GOG-0218 (Phase III)/NCT00262847 [18]	Newly diagnosed advanced epithelial ovarian, fallopian tube, or primary peritoneal cancer	Bevacizumab + Carboplatin/Paclitaxel vs. Carboplatin/Paclitaxel	PFS improved (14.1 vs. 10.3 months)	FIGO stage III (with residual disease) or IV after primary cytoreductive surgery; GOG PS 0–2; adequate organ/coagulation function; no bowel obstruction or high GI perforation risk.
ICON7 (Phase III)/NCT00483782 [19]	Newly diagnosed ovarian, fallopian tube, or primary peritoneal cancer (high-risk early stage or advanced)	Bevacizumab + Carboplatin/Paclitaxel vs. Carboplatin/Paclitaxel	PFS improved (19.0 vs. 17.3 months); greatest benefit in high-risk subgroup	High-risk early stage (I–IIA) or stage IIB–IV; prior debulking surgery or biopsy; ECOG 0–2; adequate organ function.
OCEANS (Phase III)/NCT00434642 [49]	Recurrent platinum-sensitive ovarian cancer	Bevacizumab + Carboplatin/Gemcitabine vs. Carboplatin/Gemcitabine	PFS improved (12.4 vs. 8.4 months); OS not significantly different	Recurrent disease ≥6 months after prior platinum therapy; measurable disease; ECOG 0–1; no prior bevacizumab; adequate organ function.
AURELIA (Phase III)/NCT00976911 [50]	Recurrent platinum-resistant ovarian cancer	Bevacizumab + single-agent chemotherapy (paclitaxel, PLD, or topotecan) vs. chemotherapy alone	PFS improved (6.7 vs. 3.4 months); ORR ↑	Platinum-resistant disease (<6 months since last platinum); up to two prior regimens; ECOG 0–2; no bowel obstruction or high GI perforation risk.
GOG-0213 (Phase III)/NCT00565851 [51]	Recurrent platinum-sensitive ovarian cancer	Carboplatin/Paclitaxel ± Bevacizumab	OS improved (42.2 vs. 37.3 months); PFS improved	First recurrence ≥6 months after platinum; candidates for platinum-based therapy; ECOG 0–2; adequate organ function.
MITO16B/MaNGO-OV2B (Phase III/NCT01802749 [52]	Recurrent platinum-sensitive ovarian cancer previously treated with bevacizumab	Platinum-based chemotherapy + Bevacizumab vs. chemotherapy alone	PFS improved (11.8 vs. 8.8 months)	Platinum-sensitive relapse after prior bevacizumab exposure; ECOG 0–2; adequate organ function; no contraindications to bevacizumab.

**Table 3:** TEAEs and management strategies for rucaparib according to [97,127,128], where TEAE—treatment-emergent adverse event; CBC—complete blood count; ALT—alanine aminotransferase; AST—aspartate aminotransferase; G-CSF—granulocyte colony-stimulating factor; LFTs—liver function tests; PARP—poly(ADP-ribose) polymerase; CTCAE—Common Terminology Criteria for Adverse Events.

TEAE	Frequency/Severity	Timing/ Clinical Features	Recommended Management
Nausea and Vomiting	Very common; usually grade 1–2	Early onset, often within first treatment cycles; typically manageable	Use prophylactic or as-needed antiemetics; dietary modifications; temporary dose interruption if persistent or severe.
Fatigue	Common; may be cumulative	Gradual onset during treatment; may affect daily activities	Encourage activity pacing and energy conservation; evaluate for anemia or thyroid dysfunction; dose adjustment if severe.
Anemia	Common; most frequent hematologic toxicity	Typically develops within first 8–12 weeks; may be symptomatic	Monitor CBC regularly; transfuse for symptomatic anemia; dose interruption or reduction for persistent grade ≥3 anemia.
Thrombocytopenia	Common; dose-dependent	Decline in platelet count during early or cumulative exposure	Monitor CBC routinely; hold therapy for grade ≥3 thrombocytopenia; resume at reduced dose; platelet transfusion if clinically indicated.
Neutropenia	Less common than anemia	Usually asymptomatic; risk of infection if severe	Monitor CBC and assess for infection or fever; hold therapy for grade ≥3 neutropenia; resume with dose reduction; consider G-CSF if indicated.
ALT/AST Elevation	Common; usually mild to moderate	Often early and asymptomatic; typically reversible	Monitor LFTs every 2–4 weeks initially; continue treatment for mild elevation; hold treatment for grade ≥3 and restart at reduced dose after recovery.

**Table 4:** Key clinical trials of rucaparib, where IV—intravenous; PK—pharmacokinetics; PARP—poly(ADP-ribose) polymerase; ECOG—Eastern Cooperative Oncology Group; PS—performance status; ORR—objective response rate; CR—complete response; DoR—duration of response; BID—twice daily; PFS—progression-free survival; BRCA—breast cancer susceptibility gene; HRD—homologous recombination deficiency; LOH—loss of heterozygosity; ITT—intention to treat; HR—hazard ratio; HRR—homologous recombination repair; mCRPC—metastatic castration-resistant prostate cancer; and AR—androgen receptor.

Trial	Population/Cancer Setting	Design/Combination	Key Findings	Inclusion/ Eligibility Criteria
Preclinical/early translational study [93]	Ovarian cancer cell lines and xenograft models	Rucaparib ± platinum agents	Synergistic cytotoxicity with DNA-damaging agents	Preclinical human ovarian cancer cell lines (BRCA-mutant and wild-type) and mouse xenograft models; not applicable to human eligibility.
Study 10 (Phase I/II)/NCT01482715 [86]	BRCA1/2-mutated ovarian carcinoma	Oral rucaparib 600 mg BID	ORR 59.3%; CR 10%; median duration of response ~9.7 months	Adults with high-grade ovarian carcinoma and germline or somatic BRCA1/2 mutation; ≥3 and ≤4 prior lines (BRCA cohort); ECOG PS 0–2; adequate organ function; measurable or evaluable disease.
ARIEL2 (Phase II)/NCT01891344 [29]	Platinum-sensitive ovarian cancer (BRCA/HRD stratified)	Oral rucaparib 600 mg BID	Median PFS: 12.8 mo (BRCA-mut), 5.7 mo (LOH-high), 5.2 mo (LOH-low)	Relapsed platinum-sensitive high-grade serous ovarian, fallopian tube, or primary peritoneal cancer; measurable disease; prior platinum response; tumor tissue available for genomic testing; ECOG 0–2.
ARIEL3 (Phase III)/NCT01968213 [31]	Platinum-sensitive relapsed high-grade ovarian cancer	Rucaparib 600 mg BID vs. placebo (maintenance)	PFS improved across BRCA-mutant, HRD-positive, and ITT populations	Adults with recurrent platinum-sensitive high-grade serous or endometrioid ovarian, fallopian tube, or primary peritoneal cancer responding to most recent platinum therapy; ECOG 0–2; adequate organ function; biomarker status documented.
ARIEL4 (Phase III, confirmatory)/NCT02855944 [100]	Relapsed BRCA-mutated ovarian cancer	Rucaparib vs standard chemotherapy	PFS benefit with rucaparib (HR 0.64)	Adults with high-grade serous or grade 2–3 endometrioid ovarian, fallopian tube, or primary peritoneal cancer; BRCA mutation; ≥2 prior chemotherapy regimens; adequate organ function; ECOG PS per protocol.
ATHENA-MONO (Phase III)/NCT03522246 [101]	Newly diagnosed advanced ovarian cancer	Rucaparib vs placebo (first-line maintenance)	Significant PFS improvement in HRD-positive and ITT populations; established frontline maintenance activity	Stage III–IV high-grade ovarian, fallopian tube, or primary peritoneal cancer; response (CR/PR) after first-line platinum-based chemotherapy; ECOG 0–1; no prior PARP inhibitor
ATHENA-COMBO (Phase III)/NCT03522246 [102]	Newly diagnosed advanced ovarian cancer	Rucaparib + nivolumab vs. rucaparib alone (first-line maintenance)	Addition of nivolumab did not significantly improve PFS over rucaparib monotherapy	Same frontline eligibility as ATHENA-MONO; adequate organ function; no prior immune checkpoint inhibitor
MAMOC/NOGGO Ov-42 (Phase III)/NCT04227575 [103]	Frontline ovarian cancer after bevacizumab-containing therapy	Rucaparib vs placebo (maintenance after bevacizumab)	Evaluates benefit of rucaparib following bevacizumab maintenance; results pending/ongoing in many reviews	Advanced high-grade ovarian cancer; prior carboplatin-based chemotherapy with bevacizumab; BRCA-wild-type population emphasized; response to first-line therapy; ECOG 0–1
Early-phase combination studies (Phase I/II)/NCT02354131 [104]	Recurrent ovarian cancer	Rucaparib + bevacizumab (dose-finding/safety)	Demonstrated manageable safety and biological rationale for PARP–antiangiogenic combinations	Recurrent ovarian cancer; prior platinum exposure; adequate hematologic and organ function

**Table 5:** TEAEs and management strategies for mirvetuximab soravtansine according to [33,132,135,151], where TEAE—treatment-emergent adverse event; CBC—complete blood count; AST—aspartate aminotransferase; ALT—alanine aminotransferase; and 5-HT3—5-hydroxytryptamine type 3 (serotonin) receptor.

TEAE	Frequency/Severity	Timing/ Clinical Features	Recommended Management
Blurred vision/visual disturbance	Very common (≈40% all grades); grade ≥3 ~5%	Median onset 5–7 weeks; blurred vision, visual acuity changes	Baseline ophthalmologic assessment; patient education; regular slit-lamp examinations; prophylactic lubricating eye drops; treatment interruption and dose reduction for ≥grade 2 events; ophthalmology referral.
Keratopathy/corneal epitheliopathy	Common (≈30% all grades); grade ≥3 ~5%	Median onset 5–7 weeks; corneal epithelial changes, dry eye symptoms	Baseline and periodic ophthalmologic exams; prophylactic lubrication; temporary treatment hold until improvement; topical corticosteroids if indicated under ophthalmology supervision.
Nausea/vomiting	Common (≈40%); grade ≥3 <5%	Early onset (days to weeks)	Antiemetic prophylaxis (5-HT3 antagonist ± dexamethasone); hydration and dietary support; dose modification for persistent or severe symptoms.
Diarrhea	Common (≈40%); usually low-grade	Early onset (days to weeks)	Early initiation of antidiarrheals (e.g., loperamide); maintain hydration; exclude infectious causes; temporary treatment hold for grade ≥2 events.
Fatigue/asthenia	Common (≈35%); grade ≥3 ~15–18%	Develops over weeks; may be cumulative	Supportive care and energy conservation; evaluate for anemia or thyroid dysfunction; dose interruption or reduction for persistent grade ≥3 fatigue.
Hematologic toxicities (anemia, neutropenia)	Common; anemia ≈22–28%; grade ≥3 in minority	Develops over weeks	Regular CBC monitoring; transfusion or growth-factor support as clinically indicated; dose modification per protocol for cytopenias.
Elevated liver enzymes (AST/ALT)	Occasional; grade ≥3 rare	Develops over weeks; usually asymptomatic	Periodic liver function monitoring; temporary dose hold for persistent elevation; evaluate for alternative hepatotoxic causes.
Treatment discontinuation due to TEAEs	~12% overall	Most often related to ocular toxicity or fatigue	Individualized management based on severity and recovery; ophthalmologic follow-up for visual toxicity; permanent discontinuation if clinically indicated.

Ocular toxicities (keratopathy, blurred vision) are characteristic and dose-limiting for DM4-containing ADCs. Median time to onset is typically 5–7 weeks but can vary, and early ophthalmic monitoring plus prophylactic lubrication reduces severity and supports continuation of therapy when possible. For all toxicities, follow local protocol/product labeling for specific dose-hold and dose-reduction criteria.

**Table 6:** Major clinical trials of mirvetuximab soravtansine, where MIRV—mirvetuximab soravtansine; FRα—folate receptor alpha; PK—pharmacokinetics; MTD—maximum tolerated dose; AIBW—adjusted ideal body weight; ECOG—Eastern Cooperative Oncology Group; PS—performance status; RECIST—Response Evaluation Criteria in Solid Tumors; ORR—objective response rate; DOR—duration of response; PFS—progression-free survival; OS—overall survival; PFI—platinum-free interval; IHC—immunohistochemistry; CR—complete response; PR—partial response; SD—stable disease; FDA—U.S. Food and Drug Administration; and PD-1—programmed death-1.

Trial	Population/Cancer Setting	Design/Combination	Key Findings	Inclusion/ Eligibility Criteria
Phase I Dose Escalation/(NCT01609556) [33]	Recurrent ovarian cancer	First-in-human MIRV monotherapy; safety, PK, MTD	Established recommended dose 6 mg/kg AIBW; manageable toxicity	Adults ≥18 with recurrent epithelial ovarian, primary peritoneal, or fallopian-tube cancer; measurable/evaluable disease (RECIST); ECOG PS 0–2; adequate hematologic, hepatic, and renal function; prior therapies allowed per cohort; informed consent.
FORWARD II (Phase Ib/II)/NCT02606305 [148,149]	FRα-positive ovarian cancer (platinum-resistant and platinum-sensitive)	MIRV combinations (with bevacizumab, pembrolizumab, or carboplatin); safety and ORR	Active; expansion cohorts reported; promising activity signals	Adults ≥18 with recurrent epithelial ovarian, primary peritoneal, or fallopian-tube cancer; FRα expression required (central IHC; cohort-specific cutoffs); ECOG 0–2; measurable disease; adequate organ function; prior therapies per combination arm.
MIRV + Pembrolizumab (Phase Ib/II)/NCT03835819 [162]	FRα-positive ovarian and endometrial cancers	MIRV plus anti-PD-1 therapy; safety and ORR	Early-phase cohorts completed; expansion ongoing	Adults ≥18 with advanced/recurrent FRα-positive ovarian cancer or advanced/recurrent endometrial cancer per protocol; measurable disease (RECIST 1.1); ECOG 0–1/2 (per cohort); adequate organ function; no active autoimmune disease requiring immunosuppression.
FORWARD I (Phase III)/NCT02631876 [144]	Platinum-resistant ovarian cancer (FRα medium/high)	MIRV vs investigator’s-choice chemotherapy	No overall PFS/OS benefit; PFS improvement in FRα-high subgroup (6.7 vs. 3.9 months)	Women ≥18 with epithelial ovarian, primary peritoneal, or fallopian-tube cancer; platinum-resistant disease (PFI ≤6 months); FRα medium/high by central IHC; ECOG 0–2; measurable disease; ≤3 prior regimens.
PICCOLO (Phase II)/NCT05041257 [163]	FRα-positive, platinum-sensitive ovarian cancer (≥3rd line)	MIRV monotherapy; safety and ORR	Enrollment complete; final analysis pending	Adults ≥18 with recurrent epithelial ovarian, primary peritoneal, or fallopian-tube cancer; FRα-positive by central IHC; platinum-sensitive relapse; ≥2 prior lines of therapy; ECOG 0–2; measurable disease; adequate organ function.
SORAYA (Phase II)/NCT04296890 [33]	FRα-high, platinum-resistant ovarian cancer (1–3 prior lines)	MIRV monotherapy; ORR primary endpoint	ORR 32.4%; median DOR 6.9 months; FDA accelerated approval	Women ≥18 with high-grade serous epithelial ovarian, primary peritoneal, or fallopian-tube cancer; platinum-resistant disease; FRα-high by central IHC; measurable disease; ECOG 0–1/2; no prior MIRV.
MIRV + Bevacizumab (Pooled Analysis) [147]	FRα-positive solid tumors (predominantly ovarian)	Pooled analysis of MIRV + bevacizumab	ORR 43%; median PFS 7.8 months; improved outcomes vs. MIRV alone	Adults with FRα-positive tumors enrolled across contributing studies; measurable disease; ECOG 0–2; adequate organ function; eligibility varied by parent trial.
GLORIOSA (Phase III)/NCT05445778 [150]	FRα-high platinum-sensitive ovarian cancer (maintenance after 2L platinum + bevacizumab)	MIRV + bevacizumab vs. bevacizumab alone (maintenance)	Recruiting; ongoing	Women ≥18 with high-grade epithelial ovarian, primary peritoneal, or fallopian-tube cancer; FRα-high by central IHC; response or stable disease after second-line platinum + bevacizumab; ECOG 0–1/2; no prior MIRV.
MIRASOL (Phase III)/NCT04209855 [34,153]	FRα-high platinum-resistant ovarian cancer	MIRV vs investigator’s-choice chemotherapy	Improved PFS (5.6 vs. 4.0 mo), ORR (42.3% vs. 15.9%), and OS (16.5 vs. 12.8 mo); met all endpoints	Adults ≥18 with high-grade epithelial ovarian, primary peritoneal, or fallopian-tube cancer; platinum-resistant disease; FRα-high by central Ventana FOLR1 RxDx assay; 1–3 prior regimens; ECOG 0–1/2; no prior MIRV.

**Table 7:** Contemporary management of gynecologic malignancies — ovarian cancer: current methods of treatment, where ADC—Antibody-drug conjugate, AUC—area under the concentration–time curve (chemotherapy dosing unit), BRCA—breast cancer susceptibility gene (germline or somatic), dMMR—deficient mismatch repair, FRα—folate receptor-alpha, HRD—homologous recombination deficiency, HTN—hypertension, ICI—immune checkpoint inhibitor, IDS—interval debulking surgery, IP—intraperitoneal, MDS/AML—myelodysplastic syndrome/acute myeloid leukemia, NACT—neoadjuvant chemotherapy, OS—overall survival, PARP—poly(ADP-ribose) polymerase, PDS—primary debulking surgery, PFI—platinum-free interval, PFS—progression-free survival, PLD—pegylated liposomal doxorubicin, PROC—platinum-resistant ovarian cancer, and VEGF—vascular endothelial growth factor.

Modality	Indication/ When Used	Example Regimens/ Agents	Key Evidence	Biomarkers/ Toxicity
Primary Cytoreductive Surgery (PDS)	Newly diagnosed advanced disease when optimal cytoreduction achievable	Maximal surgical debulking to no gross residual disease	Complete cytoreduction improves survival [213,214]	Prognosis strongly linked to residual tumor; requires specialized gynecologic oncology team
Neoadjuvant Chemotherapy (NACT) → Interval Debulking Surgery (IDS)	Used when PDS unlikely or patient has high surgical risk	Carboplatin + Paclitaxel (3 cycles) → IDS → further chemotherapy	NACT non-inferior to upfront PDS in selected patients [215,216]	Reduces perioperative morbidity vs. PDS in high-burden disease
First-line Platinum-based Chemotherapy	Standard systemic treatment after surgery or before IDS	Carboplatin (AUC 5–6) + Paclitaxel (175 mg/m^2^ q3w)	Standard of care per NCCN/ESMO [8,213,217]	Monitor for neuropathy, myelosuppression
Bevacizumab (Anti-VEGF) with Chemo ± Maintenance	Higher-risk advanced disease	Carboplatin + Paclitaxel + Bevacizumab → maintenance	Improves progression-free survival (PFS) [218,219]	Hypertension, proteinuria, thrombosis, GI perforation
PARP Inhibitors (Maintenance)	Platinum responders; greatest benefit in BRCA-mutated/HRD-positive tumors	Olaparib, Niraparib, Rucaparib	SOLO-1 (BRCA+) [220]; PAOLA-1 (HRD+) [27]; PRIMA (all-comers) [221]; ARIEL3 (recurrent) [30]	Requires BRCA/HRD testing; hematologic toxicity; rare MDS/AML
Mirvetuximab Soravtansine (ADC)	Platinum-resistant, FRα-high ovarian cancer	Mirvetuximab soravtansine monotherapy	SORAYA and MIRASOL trials demonstrated benefit [34,222]	Requires FRα expression testing; notable ocular toxicity
HIPEC at IDS	Selected stage III patients undergoing IDS after NACT	Cisplatin HIPEC during surgery	OVHIPEC: improved OS and PFS [223]	Use in experienced centers only; increased operative complexity
Intraperitoneal (IP) Chemotherapy	Selected stage III patients after optimal debulking	IP cisplatin-based regimens	Improved outcomes but high toxicity and catheter complications [224]	Declining use; highly center-specific
Recurrent Disease Therapy	Based on platinum-free interval	Platinum-sensitive: re-platinum ± PARPi; Platinum-resistant: PLD, weekly paclitaxel, topotecan, mirvetuximab sorvatansine	Treatment individualized by platinum sensitivity pathways [8,213]	Targeted therapy guided by BRCA/HRD/FRα
Immunotherapy (ICIs)	Standard only for MSI-H/dMMR tumors; otherwise trials	Pembrolizumab, nivolumab (biomarker-selected)	Limited benefit in unselected populations [225]	Test for MSI-H, dMMR, TMB-high
Palliative Care	Throughout disease course	Symptom control, paracentesis, pain management, psychosocial support	Improves quality of life and may prolong survival [8]	Should begin early, not limited to end-of-life

Test tumors early for germline/somatic BRCA and HRD status and other actionable markers (FRα) because the results guide the use of PARP inhibitors and targeted ADCs. Surgery vs. NACT is individualized — PDS remains standard when complete cytoreduction is achievable. NACT + IDS is appropriate when optimal upfront surgery is unlikely. PARP inhibitor maintenance (olaparib, niraparib, rucaparib) is now a central part of the first-line strategy for biomarker-selected patients and improves progression-free (and in subsets overall) survival; bevacizumab remains an option in the first line and at relapse and can be combined with PARP inhibitors in selected HRD-positive patients according to guideline pathways. HIPEC at interval cytoreduction has demonstrated survival benefit in selected stage III patients in randomized data and can be considered at experienced centres.

## Data Availability

No new data were created or analyzed in this study. Data sharing is not applicable to this article.

## Abbreviations

The following abbreviations are used in this manuscript:

2L	Second-Line
5-HT3	5-Hydroxytryptamine Type 3 Receptor
ACE	Angiotensin-Converting Enzyme
ADC	Antibody–Drug Conjugate
AIBW	Adjusted Ideal Body Weight
ALT	Alanine Aminotransferase
AR	Androgen Receptor
ASCO	American Society of Clinical Oncology
AST	Aspartate Aminotransferase
ATE	Arterial Thromboembolic Event
ATHENA-MONO	ATHENA Trial Rucaparib Monotherapy Arm
ATHENA-COMBO	ATHENA Trial Rucaparib + Nivolumab Arm
AUC	Area Under the Concentration–Time Curve
AURELIA	Platinum-Resistant Ovarian Cancer Trial
BID	Twice Daily
BP	Blood Pressure
BRCA	Breast Cancer Susceptibility Gene (BRCA1/BRCA2)
CA-125	Cancer Antigen 125
Carbo	Carboplatin
CBC	Complete Blood Count
CCB	Calcium Channel Blocker
CNS	Central Nervous System
CP	Carboplatin/Paclitaxel
CR	Complete Response
CT	Computed Tomography
CTCAE	Common Terminology Criteria for Adverse Events
DM4	N2′-Deacetyl-N2′-(3-Mercapto-1-Oxopropyl)-Maytansine
dMMR	Deficient Mismatch Repair
DoR/DOR	Duration of Response
DVT	Deep Vein Thrombosis
EC	Endometrial Cancer
ECOG	Eastern Cooperative Oncology Group
ENGOT	European Network of Gynaecological Oncological Trial Groups
EOC	Epithelial Ovarian Cancer
FDA	U.S. Food and Drug Administration
FIGO	International Federation of Gynecology and Obstetrics
FRα	Folate Receptor Alpha
Folate Receptor Alpha	Fallopian Tube
G2–M	G2 to M Phase Cell-Cycle Transition
GBM	Glioblastoma
G-CSF	Granulocyte–Colony Stimulating Factor
Gem	Gemcitabine
GI	Gastrointestinal
GLORIOSA	MIRV Maintenance Study
GOG	Gynecologic Oncology Group
GOG-0213	Platinum-Sensitive Recurrent Ovarian Cancer Trial
GOG-0218	Frontline Bevacizumab Trial in Ovarian Cancer
HCC	Hepatocellular Carcinoma
HE4	Human Epididymis Protein 4
HGSOC	High-Grade Serous Ovarian Cancer
HIPEC	Hyperthermic Intraperitoneal Chemotherapy
HR	Hazard Ratio
HRD	Homologous Recombination Deficiency
HRR	Homologous Recombination Repair
HTN	Hypertension
ICI	Immune Checkpoint Inhibitor
ICON(7)	International Collaboration on Ovarian Neoplasms (Trial 7)
IDS	Interval Debulking Surgery
IFL	Irinotecan, 5-Fluorouracil, Leucovorin
IFN-α	Interferon Alpha
IHC	Immunohistochemistry
IO	Immunotherapy/Immuno-Oncology
IP	Intraperitoneal (Chemotherapy)
ITT	Intention-to-Treat
IV	Intravenous
KPS	Karnofsky Performance Status
LFT	Liver Function Test
LGSC	Low-Grade Serous Carcinoma
LOH	Loss of Heterozygosity
MaNGO	Mario Negri Gynecologic Oncology Group
MAMOC/NOGGO Ov-42	Rucaparib Maintenance After Bevacizumab First-Line Therapy Study
MAVOC 7V-FA	PARP Inhibitor Maintenance After Prior Bevacizumab Study
mCRPC	Metastatic Castration-Resistant Prostate Cancer
MDS/AML	Myelodysplastic Syndrome/Acute Myeloid Leukemia
MI	Myocardial Infarction
MIRASOL	MIRV vs Chemotherapy in FRα-High Platinum-Resistant Ovarian Cancer
MIRV	Mirvetuximab Soravtansine
MITO	Multicenter Italian Trials in Ovarian Cancer
MITO16B	Italian Multicenter Trial in Platinum-Sensitive Recurrence
Mo	Months
MSI-H	Microsatellite Instability–High
MTD	Maximum Tolerated Dose
NACT	Neoadjuvant Chemotherapy
NOGGO	North-Eastern German Society of Gynecological Oncology
NSCLC	Non–Small-Cell Lung Cancer
OC	Ovarian Cancer
OCEANS	Platinum-Sensitive Recurrent Ovarian Cancer Trial
ORR	Objective Response Rate
OS	Overall Survival
Pac	Paclitaxel
PAOLA-1	Olaparib + Bevacizumab First-Line Maintenance Study
PARP(i)	Poly(ADP-Ribose) Polymerase (Inhibitor)
PD-1	Programmed Death Protein 1
PDS	Primary Debulking Surgery (Primary Cytoreductive Surgery)
PE	Pulmonary Embolism
PFI	Platinum-Free Interval
PFS	Progression-Free Survival
PK	Pharmacokinetics
PLD	Pegylated Liposomal Doxorubicin
PR	Partial Response
PRIMA	Niraparib First-Line Maintenance Study
PROC	Platinum-Resistant Ovarian Cancer
PS	Performance Status
QoL	Quality of Life
RCC	Renal Cell Carcinoma
RECIST	Response Evaluation Criteria in Solid Tumors
RT	Radiotherapy
SD	Stable Disease
SGO	Society of Gynecologic Oncology
SOLO-1	Study of Olaparib in First-Line Maintenance
SORAYA	MIRV in FRα-High Platinum-Resistant Ovarian Cancer
TEAE	Treatment-Emergent Adverse Event
TMZ	Temozolomide
VEGF	Vascular Endothelial Growth Factor
